# Porous silicon nanoparticles for cancer photothermotherapy

**DOI:** 10.1186/1556-276X-6-321

**Published:** 2011-04-11

**Authors:** Chanseok Hong, Jungkeun Lee, Hongmei Zheng, Soon-Sun Hong, Chongmu Lee

**Affiliations:** 1 Department of Materials Science and Engineering, Inha University, 253 Yonghyeon-dong, Incheon, 402-751, Republic of Korea; 2 College of Medicine, Inha University, Shinheong-dong, Incheon, 400-712, Republic of Korea

## Abstract

The *in vitro *cell tests and *in vivo *animal tests were performed to investigate the feasibility of the photothermal therapy based on porous silicon (PSi) in combination with near-infrared (NIR) laser. According to the Annexin V- fluorescein isothiocyanate Apoptosis assay test results, the untreated cells and the cells exposed to NIR laser without PSi treatment had a cell viability of 95.6 and 91.3%, respectively. Likewise, the cells treated with PSi but not with NIR irradiation also had a cell viability of 74.4%. Combination of these two techniques, however, showed a cell viability of 6.7%. Also, the cell deaths were mostly due to necrosis but partly due to late apoptosis. The *in vivo *animal test results showed that the Murine colon carcinoma (CT-26) tumors were completely resorbed without nearly giving damage to surrounding healthy tissue within 5 days of PSi and NIR laser treatment. Tumors have not recurred at all in the PSi/NIR treatment groups thereafter. Both the *in vitro *cell test and *in vivo *animal test results suggest that thermotherapy based on PSi in combination with NIR laser irradiation is an efficient technique to selectively destroy cancer cells without damaging the surrounding healthy cells.

## Introduction

In recent years, photothermotherapy (PTT) techniques based on inorganic nanomaterials and near-infrared (NIR) light have attracted significant attention owing to their advantages over conventional surgical treatments. The advantages of PTT include the anticipated reduction in morbidity and mortality, low cost, suitability for real-time imaging guidance, and the ability to perform ablative procedures on outpatients because of its non-invasive nature [1]. In the conventional PTTs based on simple heating, i.e., hyperthermia [[2], and references therein], most treatment failures result from insufficient temperature rises in the tumor tissues. Therefore, it is essential to use a thermal coupling agent with a good photothermal property to secure irreversible destruction of tumor cells in a short time without damaging adjacent healthy cells in thermotherapy.

The inorganic nanomaterials currently demonstrated as thermal coupling agents in PTTs are gold nanoparticles (Au NPs) [3-12], gold nanorods [13-16], gold nanoshells [1,17-19], gold nanocages [20,21], gold nanocrystals [22,23], single wall carbon nanotubes (SWCNTs) [24-26], and porous silicon (PSi) [27,28]. Of these nanomaterials, we are particularly interested in PSi because it is known to have many important properties such as biocompatibility [29], biodegradability [30,31], and a readily functionalized surface [32] which a therapeutic agent should have desirably as well as an excellent photothermal property [33]. In addition, PSi has a merit that it can be easily prepared by the simple electrochemical anodization of silicon. As regards biomedical applications of PSi, their use in drug delivery [34-39] and photodynamic therapy [40-42] applications have been reported before. We previously reported on the excellent heat generation ability of PSi and the ability of PSi to irreversibly destroy cancer cells under NIR laser irradiation by using temperature rise measurement and MTT assay results, respectively [27,28]. In this paper, we report the Annexin V-fluorescein isothiocyanate (FITC) apoptosis assay test and *in vivo *animal test results of PSi in combination with NIR laser to investigate the ability of PSi to kill cancer cells as well as the death modes of cancer cells and the ability of PSi to inhibit the growth of tumors, respectively.

## Experimental details

### Preparation of PSi/EtOH:PEG drug solutions

First, meso-PSi layers were prepared on 2.5 cm × 2.5 cm × 0.05 cm pieces of p-type Si(100) with a resistivity of 1-5 mΩcm by anodic etching in an 3:1 (by volume) solution of 46% HF and 95% C_2_H_5_OH at a current density of 200 mA/cm^2 ^for 150 s. PSi is generally classified into three different types in terms of the pore size: macro-PSi (*d *> 50 nm), meso-PSi (2 nm <*d *< 50 nm), micro-PSi (*d *< 2 nm). According to our experience, meso-PSi is the most suitable for photothermotherapy since it shows the highest photothermal effect and can be easily to be fractured into nanoparticles with proper sizes. The porosity and thickness of the PSi layers determined by weight measurements [43] were about 73% and 55 μm, respectively. The details of the anodization process are described elsewhere [27]. The PSi layers formed on Si(100) were then lifted off by anodic etching in an 1:15 (by volume) solution of 46% HF and 95% C_2_H_5_OH at a current density of 4 mA/cm^2 ^for 250 s. Next, the free-standing PSi layers were fractured by ultrasonicating in 10 mL of ethanol for 24 h. The PSi nanoparticles were subsequently filtered twice by using a 450 nm membrane first and then by using a 220 nm membrane. PSi/EtOH:PEG drug solutions were prepared by dispersing the resulting PSi particles in 10 mL of ethanol mixed with 10 mL of thiolated polyethyleneglycol (PEG-SH) and centrifuged for 24 h until all the PSi particles were dispersed.

### Measurement of heating of the PSi/EtOH:PEG solution by NIR irradiation

Heterochromatic NIR light irradiation was performed on six different samples by using NIR laser. The samples include a PSi/EtOH:PEG solution and a EtOH:PEG solution as well as a solid PSi (a free standing PSi layer). The three different kinds of samples were irradiated continuously for 20 min by the NIR laser at 1.5 W/cm^2^. The distance between the laser source and each sample was fixed to be 2 cm. Change in the temperature of the samples with the NIR exposure time was measured at 30-s intervals by using an IR thermometer (model: AZ 8859, max. output: 1 mW, wavelength: 670 nm, measurement range: -20 to 420°C).

### Cells and materials

The Murine colon cancer cell lines (CT-26) were purchased from the Korean Cell Line Bank (KCLB, Seoul, Korea). CT-26 cells were cultured in Dulbecco's modified Eagle's medium (DMEM), supplemented with 10% fetal bovine serum (FBS) and 1% penicillin/streptomycin. FBS, cell culture media, penicillin-streptomycin and all other agents used in cell culture studies were purchased from Invitrogen™ (GIBCO, NY, USA). Cultures were maintained at 37°C in a CO_2 _incubator with a controlled humidified atmosphere composed of 95% air and 5% CO_2_. Trypan blue dyes were purchased from Sigma-Aldrich (St. Louis, MO, USA).

### Annexin V- FITC apoptosis assays

CT-26 cells were cultured in DMEM. The incubations of 1 × 10^6 ^CT-26 cells were carried out at 37°C and in 5% CO_2 _atmosphere for approximately 24 h in 24-well plates, with the cells having been seeded in a 100 nm dish for approximately 18 h before incubation. After incubation, the cell media were removed from the wells, and the cells were washed using PBS and then incomplete DMEM was added to each well. Then, the PSi/EtOH:PEG solution was added to each well. Annexin V- FITC apoptosis assays were performed on four different mouse CT-26 cell sample groups to see the ability of our technique to irreversibly destroy cells and the modes of cell deaths: the CT-26 cell control group given neither PSi nor laser treatment, the CT-26 cell group not treated with PSi but with laser, the group not treated with laser but with PSi, the group treated with both PSi and laser. For the preparation of the last sample group, CT-26 cells were treated with the PSi/EtOH:PEG drug solution (0.7 g/L) first and then NIR laser at 600 mW/cm^2 ^for 20 min. Next, the 2 × 10^6 ^cells were removed from the culture, washed twice with cold PBS, and double-stained with Annexin V-FITC and propidium iodide (PI) (BD Biosciences, San Jose, CA, USA) in Annexin-binding buffer, followed by analysis on a FAC-Scalibur flow cytometer (Becton Dickinson, San Jose, CA, USA) equipped with a 488-nm argon laser. To avoid nonspecific fluorescence from dead cells, live cells were gated using forward and side scatter.

### Trypan blue cell death assays

Cell viability was performed by the trypan blue cell death assay. Briefly, CT-26 cells were plated at a density of 3 × 10^5 ^cells in 60 mm culture dishes for 24 h. Then, the medium was removed, and the cells were treated with a PSi/EtOH:PEG solution to each plate. The final concentration of ethanol in the medium was ≤0.5% (v/v). Detached CT-26 cells treated with a PSi/EtOH:PEG solution were exposed to NIR laser at 600 mW/cm^2 ^for 20 min. After NIR irradiation, a collection of supernatants and adherent cells obtained by trypsinization was incubated in 0.4% trypan blue and pipetted onto a hemocytometer and manually counted under a microscope at ×100 magnification. The percentage of cells admitting trypan blue dye to the total number of cells was determined by counting three different fields for each experimental condition, which was done in triplicates [44].

### *In vivo *animal tests

Animal care and all experimental procedures were conducted in accordance with the Guide for Animal Experiments edited by the Korean Academy of Medical Sciences. The CT-26 cells (1 × 10^6 ^cells) were suspended in 100 μL PBS, and subcutaneously injected into the back of male mice of each group (*n *= 5, 5- to 6-week-old, Balb/c). When the tumors were grown up to a volume of 65-70 mm^3^, mice were randomized into four groups: (a) mice were simply monitored without any other treatment; (b) mice were intratumorally injected with 100 mL of PBS and then irradiated NIR laser 4 times at 1.5 W/cm^2 ^for 2 min each time with a time interval of 2 min under the ether anesthesia; (c) a PSi/EtOH:PEG solution (0.7 g/L, 100 μL) was intratumorally injected without NIR laser irradiation; (d) a PSi/EtOH:PEG (0.7 g/L, 100 μL) was injected into tumor, then NIR laser was immediately irradiated on the tumor region in the same manner as in (a). The mice were anesthetized by injecting 40 μL of a 9:1 solution of ketamine (100 mg/mL) and rompun (100 mg/mL). Tumor volumes, animal body weights, and tumor conditions were recorded weekly for the duration of the study [45]. The tumor size of each group was measured using a skinfold caliper, and tumor volumes were calculated using the following equation: tumor volume = *ab*^2^/2, where *a *is the maximum diameter of tumor and *b *is the minimum diameter of tumor [46]. All the procedures for *in vivo *experiments were performed in accordance with Inha University of Biomedical Science guidelines on animal care and use.

## Results and discussion

### Photothermal properties of the PSi/EtOH:PEG solution

**Figure 1:**
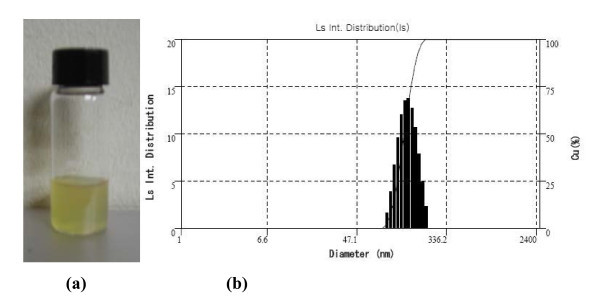
**PSi particle size distribution**: **(a) **PSi/EtOH:PEG solution and **(b) **the distribution of the diameter of PSi nanoparticles in the PSi/EtOH:PEG solution.

**Figure 2:**
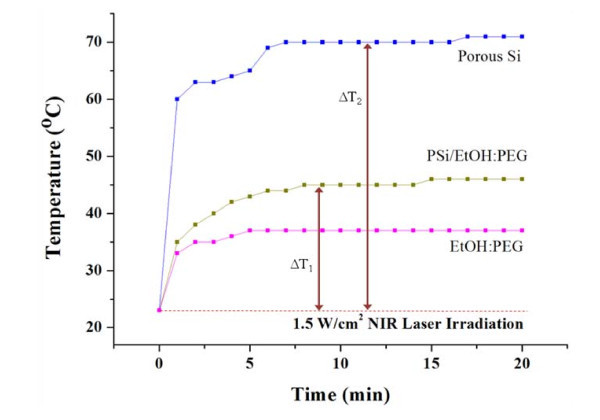
**Comparison of photothermal property between solid PSi, PSi/EtOH:PEG solution (0.7 g/L), and EtOH:PEG solution**. Δ*T*_1 _and Δ*T*_2_, respectively, in this graph represent the temperature rises in solid PSi (a free-standing PSi layer) and PSi/EtOH:PEG solution upon NIR laser irradiation at 1.5 W/cm^2^.

The PSi nanoparticles functionalized with PEG were well solublized, so that they were uniformly distributed in the PSi/EtOH:PEG solution without forming any floating particles, precipitates, or agglomerates for a long period of time as shown Figure 1a. Functionalization of PSi with PEG is necessary to enhance the internalization of PSi particles into cells as well as the attachment of antibodies to PSi particles for the systematic administration of cancer. Ethyl alcohol was also used to enhance the dispersion of PSi nanoparticles in the solution. Figure 1b displays the size distribution of the PSi nanoparticles after being filtered by using a 220 nm membrane, which was in a range from 80-220 nm in diameter with a mean diameter of approximately 140 nm. The photothermal property was compared between solid PSi, PSi/EtOH:PEG solution (0.7 g/L), and EtOH:PEG solution in Figure 2. The surface temperature of each sample during exposure to 808 nm NIR laser at 1.5 W/cm^2 ^was measured by using an IR thermometer. The PSi nanoparticles exhibited a very rapid increase in temperature for the first 1 min and a slow increase for the next 5-6 min. The temperature reached at approximately 70°C in about 6 min upon NIR laser irradiation and did not nearly changed thereafter. The net temperature rises shall have been 47°C, taking the temperatures of the PSi film at the exposure time of 0 min (~23°C) into consideration. This high photothermal effects in PSi is mainly attributed to the high absorbance and the high surface-to-volume ratio due to the numerous micropores in PSi [33]. The temperature of the PSi/EtOH:PEG solution changed with the NIR exposure time in a pattern similar to the solid PSi, but the temperature was much lower than that of the PSi nanoparticles presumably due to the absorption of heat by the liquid phases such as EtOH and PEG in the solution. The temperature difference between PSi/EtOH:PEG and EtOH:PEG is about 10°C for the NIR irradiation time larger than 15 min, which may seem to be somewhat small for thermotherapy. In the *in vivo *animal tests, the EtOH:PEG solution injected directly into tumors would move easily to the whole body of the mouse. Consequently, a very little amount of remnant solution would stay in the tumors. In contrast, the PSi/EtOH:PEG solution injected directly into tumors would stay in the tumors for a longer time because it has a higher viscosity than the simple EtOH:PEG solution. Therefore, there would be a big difference between the two solutions in the actual effect of destroying cancer cells.

### Annexin V- FITC apoptosis assay tests

**Figure 3:**
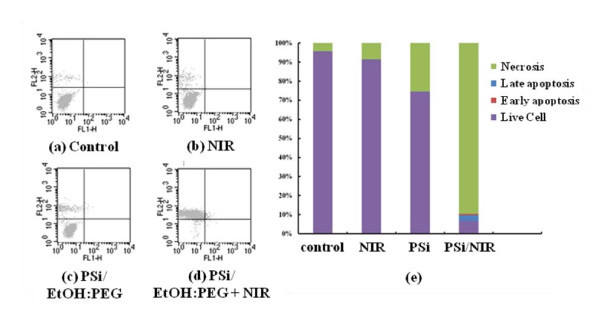
**Annexin V-FITC Apoptosis assay results**: Flow cytometry profiles represent Annexin-V-FITC staining in *X*-axis and PI in *Y*-axis: **(a) **control, i.e., neither PSi nor laser treatment (control), **(b) **PSi treatment only, **(c) **NIR laser treatment only (for 20 min at 600 mW/cm^2^), and **(d) **PSi treatment (PSi concentration = 0.7 g/L) followed by laser treatment (for 20 min at 600 mW/cm^2^). **(e) **Summary of the Annexin V-FITC Apoptosis assay results showing the percentages of cell death modes: necrosis, late apoptosis, early apoptosis, and live cell.

The fluorescent-activated cell sorter (FACS) flow cytometry profiles (Figure 3a-d) obtained as a result of Annexin V-FITC Apoptosis assay represent Annexin V-FITC staining in *X*-axis and PI in *Y*-axis. The four sections of the quadrant in each profile from the upper left in a clockwise direction represent necrosis, late apoptosis, early apoptosis, and live cell, respectively. Of these four kinds of cell modes, necrosis and late apoptosis are usually considered as cell death. The Annexin V-FITC Apoptosis assay results in (Figure 3a-d) are summarized in Figure 3e. The untreated cells and the cells exposed to NIR laser without PSi treatment had a cell viability of 95.6 and 91.3%, respectively. Likewise, the cells treated with PSi but not with NIR irradiation also had a cell viability of 74.4%. Combination of these two techniques, however, showed a cell viability of 6.7%, implying that most cells are killed. The group treated with both PSi and NIR laser shows substantially higher cell death (necrosis + late apoptosis) rate than those not given both treatments. It can also be seen in Figure 3e that the cell deaths are mostly due to necrosis but partly due to late apoptosis. This *in vitro *cell test result suggests that only combination of PSi and NIR laser treatments can kill cells. The viability of 74.4% for the cells treated with PSi but not with NIR irradiation seems to be low, suggesting that PSi is somewhat toxic. This toxicity of PSi may originate from HF residues on the surfaces of the PSi nanoparticles due to incomplete washing. More effort should be made to remove all the HF residues during the course of PSi nanoparticles preparation.

We previously reported as a result of the *in vitro *cell test based on MTT assay that the cell viabilities of the mouse groups untreated, treated only with PSi, treated only with laser, and treated with PSi followed by laser treatment were 99.8, 95.2, 98.1, and 2.6%, respectively [28]. The present Annexin V-FITC Apoptosis assay result is somewhat worse than the previous MTT assay result. In the previous test, the PSi/NaCl suspension was used instead of the PSi/EtOH:PEG solution as a PSi drug solution. Another difference is that the PSi concentration in the PSi/NaCl suspension (~10.0 g/L) was far higher than that in PSi/EtOH:PEG solution (0.7 g/L) since the PSi was not filtered by using a 220-nm membrane in the former suspension whereas PSi was filtered prior to the *in vitro *cell test in the latter solution. Therefore, the higher cell viability, i.e., the lower cell death rate of the group treated with both PSi and laser in the present test may be mainly attributed to the lower PSi concentration in the PSi drug solution.

### Trypan blue cell death assay tests

**Figure 4:**
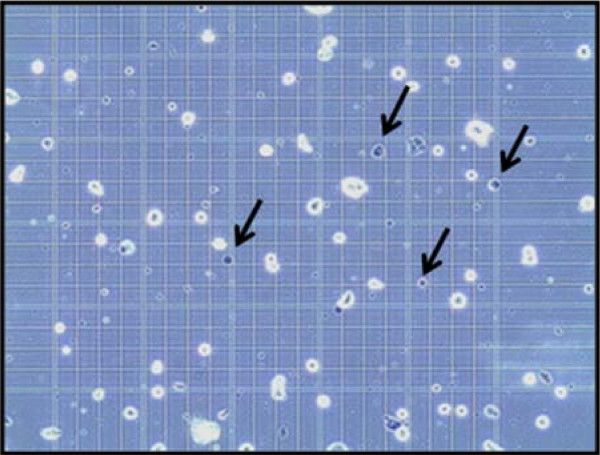
**Trypan blue staining**. Optical microscopic images of the CT-26 cells treated with a PSi/EtOH:PEG solution (0.7 g/L) followed by NIR laser treatments (for 20 min at 600 mW/cm^2^) 4 times for 2 min each time with a time interval of 2 min. The cells were stained using trypan blue dye after the NIR laser treatments to examine cell damage. The cells indicated by arrows are some examples of dead cells turned blue after staining.

To determine whether the effect of PSi nanoparticles under the NIR laser irradiation on cells was cytotoxic, trypan blue cell death assay was performed on mouse CT-26 cells to investigate localized photothermal destruction of the cancer cells. High-magnification optical microscopy images of the cells dispersed in the PSi/EtOH:PEG solution given the laser treatment is shown in Figure 4. The cells were first treated with a 0.7 g/L PSi/EtOH:PEG solution. Next, the cells were exposed to laser and then stained by trypan blue dye to examine cell damage. The color of the dead cells usually turns black after staining treatment. It can be seen in Figure 4 that more than 60% of the cells irradiated with NIR laser have turned black in color, indicating cell death.

### *In vivo *animal tests

**Figure 5:**
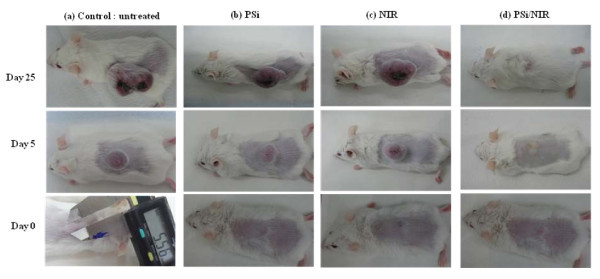
***In vivo *animal test results**. Photographs of the tumors of the mice treated differently 24 h after treatment: **(a) **neither PSi nor laser treatment (control), **(b) **PSi treatment only, **(c) **NIR laser treatment only (NIR laser irradiation 4 times for 2 min at 1.5 W/cm^2 ^each time with a time interval of 2 min), and **(d) **PSi treatment (PSi concentration = 0.7 g/L) followed by laser treatment (for 20 min at 600 mW/cm^2^).

**Figure 6:**
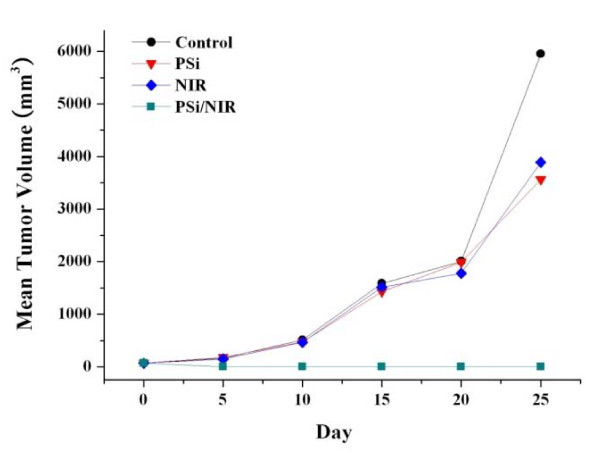
**Change of tumor volume**. Tumor volume **(a) **and ratio volume **(b) **of CT-26 tumor cell xenografts. Tumor volumes were measured once a week after sample treatments. The group treated with a PSi/EtOH:PEG solution followed by NIR laser treatments (4 times for 2 min at 1.5 W/cm^2 ^each time with a time interval of 2 min) shows efficient tumor growth inhibition compared with other experimental groups.

The *in vitro *cell test results show the photothermal effect of the thermotherapy based on PSi combined with laser on cell death, but it does not guarantee that the thermotherapy can inhibit tumor growth. To confirm that the photothermal effect of PSi combined with NIR laser could efficiently destroy tumor cells without giving damage to surrounding healthy cells, we attempted *in vivo *therapeutic examinations against Balb/c mice bearing (CT-26) on their backs. When the tumors were grown up to a volume of approximately 100 mm^3^, PSi/EtOH:PEG solution (0.7 g/L, 100 μL) were then injected directly into the tumor regions. A mouse ready for the irradiation was located under the focal lens through which NIR laser could be focused to have a power density of 1.5 W/cm^2 ^and irradiated 4 times for 2 min each time with a time interval of 2 min. This intermittent laser irradiation was designed to minimize the damage of the healthy tissues adjacent to the tumor tissues. As shown in Figure 5, the mouse treated with a PSi/EtOH:PEG solution and NIR laser irradiation shows perfect tumor destruction 25 days after the treatments. It appears that the tumor has shrinked to almost zero volume at day 5 post-treatment. The tumescent part formed on the laser-treated region is not the shrinked tumor but looks like a kind of water blister formed by thermal energy from PSi nanoparticles. Finally, the tumor has completely disappeared at day 25 post-treatment although the complete disappearance of the tumor is not clearly observable owing to the regrown fur. Comparison of the tumor site between the four groups treated differently, however, more clearly indicates the complete destruction of the tumor in the group treated both PSi and laser. It is worthy of noting that the surface structures including the epidermis and subcutaneous tissue got no damage such as carbonization at all. It is common that an esker or a black skin burn mark forms and it falls off many days after treatment in PTT. Even no such marks were observed on the mice in the group treated both PSi and laser. The intact surface structures suggest that the treatment parameters including the power intensity of the laser and the PSi concentration of the PSi/EtOH:PEG solution was adequate. It is well known that the degree of damage of the surface structures in PTT strongly depends on the treatment parameters. The mice given both PSi and laser treatments remained healthy without any recurrence of tumors and side effects for more than 3 months. In contrast, the other three groups including the control group, the group treated only with PSi, and that treated only with NIR laser show significant growth of tumors after the treatment. The size of the tumors in these groups is in a range of 1.1-1.5 cm at day 5 post-treatment. The tumors grew continuously to be 1.8-2.7 cm in diameter at day 25 post-treatment. The same kind of in vivo animal tests were repeated for another two sets of mouse groups and similar results were obtained, implying that these results are very reproducible. Figure 6 compares the tumor growth rate between the four different experimental mouse groups. The tumors in the mice treated with both PSi and laser show almost zero volume change, whereas the tumors in the other three groups continued to grow until they died (Table 1).

**Table 1:** Tumor size after PSi or laser treatment (mm)

	Control	PSi	NIR laser
Day 0	5.5 × 5.4		
Day 5	12 × 12	12 × 11	12 × 15
Day 25	21 × 27	18 × 22	18 × 24

NIR laser irradiation: 1.5 W/cm^2 ^× 2 min × 3 times

### Weight change after laser treatment

**Figure 7:**
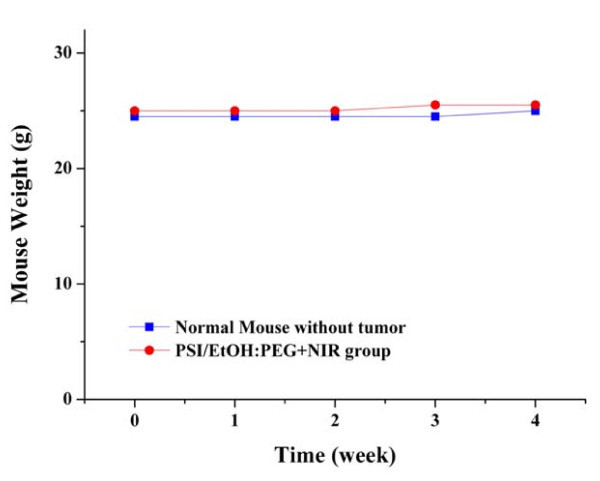
**Change of body weight**. Change in the body weight of the mice injected with a PSi/EtOH:PEG solution followed by NIR laser treatments (4 times for 2 min at 1.5 W/cm^2 ^each time with a time interval of 2 min). There is no significant body weight loss for apparent side effects.

One of the important issues in PTT using inorganic nanomaterials is the toxicity of the nanomaterials to the organs of human body. We investigated the toxicity of PSi on the organs of mouse bodies by an indirect method of measuring the change in weight after laser treatment. It is widely accepted that the animals given toxic treatment lose weight. The body weight of the mouse treated with PSi followed by laser treatment increased slightly in a pattern similar to the normal mouse without tumors (Figure 7), indicating that the mouse continued to mature without any significant toxic effect. Another important issue in PTT using inorganic nanomaterials is harmless elimination of the nanomaterials from the human body in a reasonable period of time. Park et al. [31] reported that the PSi nanoparticles used for drug delivery accumulated in the organs are noticeably cleared from the body within a period of 1 week and completely cleared in 4 weeks. The experiments on this issue are also ongoing in our lab.

## Conclusions

The *in vitro *cell test and *in vivo *animal test results were performed to investigate the feasibility of the photothermal therapy based on PSi in combination with 808 nm NIR laser. Combination of PSi and NIR laser treatment techniques shows a substantially higher cell death rate than only one of these two techniques. The Murine colon carcinoma (CT-26) tumors were completely resorbed without nearly giving damage to surrounding healthy tissue within 5 days of PSi and NIR laser treatment. All the mice given both treatments remained healthy and free of tumors and side effects for more than 3 months. The preliminary results in this work shows the feasibility of photothermotherapy based on PSi in combination with NIR laser irradiation in selectively destroying cancer cells without damaging the surrounding healthy cells. However, the systematic administration of cancers still remains as a challenge in this therapeutic approach. The experiments on this issue are under way using tumor targeting techniques such as functionalization of PSi with specific antibodies.

## Abbreviations

Au NPs: gold nanoparticles; DMEM: Dulbecco's modified Eagle's medium; FACS: fluorescent-activated cell sorter; FBS: fetal bovine serum; FITC: fluorescein isothiocyanate; NIR: near-infrared; PEG-SH: thiolated polyethyleneglycol; PSi: porous silicon; PTT: photothermotherapy; SWCNTs: single wall carbon nanotubes; PI: propidium iodide.

## Competing interests

The authors declare that they have no competing interests.

## Authors' contributions

CL carried out the cancer photothermothrapy studies, participated in their design and coordination, and drafted the manuscript. CH, JL, and HJ carried out the in vitro cell tests and in vivo animal tests. SH conceived of the study, and participated in its design and coordination. All authors read and approved the final manuscript.
